# Quantifying Charge Effects on Fouling Layer Strength and (Ir)Removability during Cross-Flow Microfiltration

**DOI:** 10.3390/membranes11010028

**Published:** 2021-01-01

**Authors:** Mads Koustrup Jørgensen, Tuve Mattsson

**Affiliations:** 1 Center for Membrane Technology, Department of Chemistry and Bioscience, Aalborg University, Fredrik Bajers Vej 7H, DK-9220 Aalborg Øst, Denmark; 2 Department of Chemistry & Chemical Engineering, Chalmers University of Technology, SE-41296 Gothenburg, Sweden; tuve.mattsson@chalmers.se; 3 Wallenberg Wood Science Center, Chalmers University of Technology, SE-41296 Gothenburg, Sweden

**Keywords:** membrane fouling, microfiltration, fouling monitoring, cake formation

## Abstract

Fouling of membranes is still an important limiting factor in the application of membrane technology. Therefore, there is still a need for an in-depth understanding of which parameters affect the (ir)removability of fouling layers, as well as the mechanisms behind fouling. In this study, fluid dynamic gauging (FDG) was used to investigate the influence of charge effects between negatively charged foulant particles and cations on cake cohesive strength. Fouling cakes’ thicknesses and cohesive strengths were estimated during membrane operations, where microfiltration (MF) membranes were fouled in a feed-and-bleed cross-flow filtration system with low and highly negatively charged polystyrene–polyacrylic acid core-shell particles. In addition, an added procedure to determine the irremovability of cakes using FDG was also proposed. Comparing layers formed in the presence and absence of calcium ions revealed that layers formed without calcium ions had significantly lower cohesive strength than layers formed in the presence of calcium ions, which is explained by the bridging effect between negatively charged particles and calcium ions. Results also confirmed more cohesive cakes formed by high negative charge particles in the presence of calcium compared to lower negative charge particles. Hence, it was demonstrated that FDG can be used to assess the cohesive strength ((ir)removability) of cake layers, and to study how cake cohesive strength depends on foulant surface charge and ionic composition of the solution.

## 1. Introduction

Membrane filtration is a flexible, compact, and energy-efficient technology widely used in applications ranging from the dairy industry [1,2,3] and breweries [4] to wastewater treatment [5] and water purification [6]. However, membrane fouling is still a major obstacle that limits further utilisation of membrane technology, because fouling results in unsteady performance and higher operating expenses (OPEX) [7,8,9]. Fouling leads to increased energy demand, chemical cleaning, membrane replacement, labour for process technicians, and thereby higher operational costs. To reduce these costs, fouling prevention is necessary, e.g., by pre-treatment (such as coagulation/flocculation) and operating at high shear stress near the surface to prevent deposition [8,9,10]. In addition, membranes are cleaned physically (backwash or relaxation) and eventually chemically [8,9,11].

Fouling can be grouped into categories of removable, irremovable, and irrecoverable fouling [8]. Removable fouling can be removed by physical cleaning, whereas chemical cleaning may degrade and remove some of the irremovable fouling. The fouling that cannot be removed by chemical and physical cleaning is denoted irrecoverable fouling.

It is evident from the literature that fouling is inevitable, hence, studies aim to control fouling by understanding the mechanisms of formation and removal and how these relate to suspension and membrane characteristics along with operating conditions [7]. Although numerous studies have investigated fouling mechanisms, there is still a need for an in-depth understanding of fouling mechanisms and which parameters affect the (ir)removability of fouling layers. This understanding may be furthered by in situ, direct measurements of fouling layer characteristics with fouling monitoring techniques [12,13,14].

To provide a direct measure of the state of the membrane, some technologies have been proposed for fouling monitoring, e.g., ultrasonic reflectometry (UR) [15,16], laser triangulometry (LT) [17], and direct observation through microscopes (DOTMs) [13,18]. All these methods can be used to estimate the thickness of fouling layers. However, optical techniques such as DOTM and LT are limited by the need for an “optical window”, hence, membrane fouling by turbid suspensions cannot be monitored. In contrast, UR can quantify fouling in a different membrane process and is not affected by feed stream turbidity [15,16,19]. However, UR relies on the difference in impedance between the fouling layer and the membrane, making the detection of organic fouling difficult [16].

Another technique is fluid dynamic gauging (FDG), which has been developed both for membrane fouling thickness monitoring and for quantifying the cohesive strength of fouling layers [20,21]. FDG utilises a probe with a nozzle which is brought close to the membrane while liquid (feed) is drawn through the nozzle. This creates an increasing pressure drop by flow constriction as the probe moves closer to a surface. By recording the pressure drop at different positions from a clean and fouled membrane, the thickness of the fouling layer can be estimated [20,22]. In addition, the resilience of the fouling layer to the fluid shear stress generated by the withdrawal of the liquid can be assessed. By advancing the probe further, fouling deposits will be removed (destructive mode operation), i.e., a scan through the fouling layer can be applied to investigate the cohesive strength throughout the layer [22,23]. The method has already been demonstrated for monitoring fouling layers formed by microfiltration (MF) of glass beads [24], yeast [20], biofilm [25], microcrystalline cellulose [12,23], lignin [26], and molasses [27], as well as layers formed during the filtration of steam explosion wood extracts [28]. Hence, fouling monitoring by FDG has been demonstrated in a range of applications and may also have the potential to monitor fouling in other applications, e.g., in the brewing, dairy, and medical industries.

In this study, it was demonstrated how FDG can be used to quantify fouling layer cohesive strength (physical irremovability) and how it depends on interactions between negatively charged foulant particles and dissolved cations. It is already established that the bridging between negatively charged foulants and bi- or polyvalent cations (e.g., Ca^2+^) increases fouling layer irremovability [29]. This study aimed not only to demonstrate this effect, but also to show how FDG can be used to quantify the effect of chemical interactions on fouling layer cohesive strength for a system where bridging between particles is present. This is done by assessing the cohesive strength of fouling layers of negatively charged particles in the presence and absence of calcium ions. In addition, an added procedure to quantify the amount of irremovable fouling is proposed, by carrying out a second FDG analysis after the first FDG analysis of a fouling layer, to determine the amount of remaining fouling after destructive mode FDG. 

Experiments were performed by carrying out cross-flow MF along with in situ FDG measurements of fouling layers’ thickness and cohesive strength. Suspensions were composed of two types of polystyrene–polyacrylic acid core-shell particles (diameter ~1.6 microns) developed in a previous study [13]; one with a low surface charge (zeta potential −28 mV) and one with a high surface charge (zeta potential −42 mV). The particles were suspended in aqueous solutions containing either sodium ions (5 mM) or sodium and calcium ions (5 mM and 7.5 mM, respectively) to impede and promote coagulation and interaction between particles in fouling layers, respectively.

## 2. Materials and Methods 

### 2.1. Microparticle Synthesis

As mentioned in the Introduction, synthetic microparticles were chosen as foulants for the fouling studies. This was because they are well defined foulants for MF, and easy to synthesise with monodisperse size distribution and configurable and reproducible surface charge [13]. Microparticles, with a narrow size distribution, consisting of polystyrene cores and polyacrylic acid shells (PS–PAA particles) were synthesised with the dispersion polymerisation method described in Lorenzen et al. [13]. The synthesis was carried out over 24 h in a nitrogen atmosphere at 70 °C in 250 mL round-bottomed flasks with magnetic stirring. Polymerisation of styrene (17.5 mL, CAS: 100-42-5, 99%, Sigma-Aldrich, St. Louis, MO, USA) was carried out in 100 mL methanol (CAS: 67-56-1, 99.8%, Sigma-Aldrich, St. Louis, MO, USA) using benzoyl peroxide (0.5 g, CAS: 94-36-0, 75% remainder water, Acros Organics, Geel, Belgium) as an initiator. The growing polystyrene chain will form a nucleus in which the polystyrene chain will continue to grow due to bulk polymerisation. Hence, particles consisting of a water-insoluble hard core forms which by further polymerisation are surrounded by a water-soluble surface layer (shell). The shell was synthesised by polymerisation of uncharged hydroxypropyl cellulose (HPC, CAS: 9003-64-2, Sigma-Aldrich, St. Louis, MO, USA) and charged polyacrylic acid (PAA, CAS: 9003-01-4, Sigma-Aldrich, St. Louis, MO, USA) in different ratios to vary surface charge. High surface charge (HC) particles were formed by adding HPC and PAA in 1:1 (wt%) ratio, whereas low surface charge (LC) particles were synthesised by adding HPC and PAA at a 3:1 (wt%) ratio. The total mass of PAA and HPC was 1.2 g. 

After synthesis, the suspensions were filtered through a 45 µm filter cloth to remove any coagulated material. Sedimented PS–PAA particles in methanol were then suspended in 5 mM NaOH after removing supernatant methanol. The replacement of supernatant with 5 mM NaOH was repeated five times to remove any unpolymerised styrene.

The zeta potential of the synthesised particles was measured with a Zetasizer from Malvern Instruments (Zetasizer nano ZS, Malvern Instruments, Malvern, UK) using a dip cell and disposable cuvette. HC particles had a zeta potential of −42 mV and LC particles −28 mV. The diameter of synthesised particles was measured on a LS 13320 particle size analyser from Beckman Coulter (Brea, Ca). The suspensions of highly and lowly charged particles were all monodisperse with similar diameters HC: 1.63 ± 0.22 µm and LC: 1.56 ± 0.23 µm.

### 2.2. FDG Cross-Flow Filtration Rig

**Figure 1:**
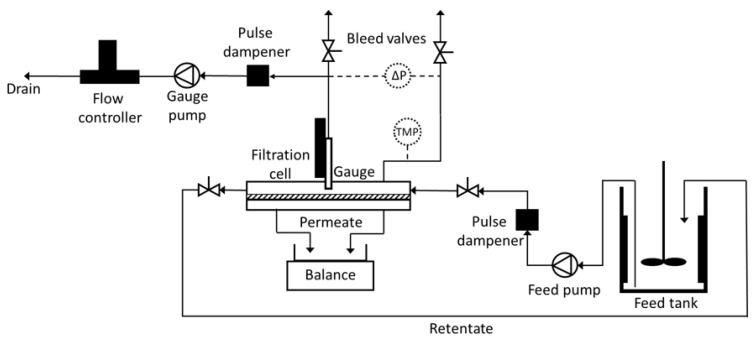
Schematic diagram of the fluid dynamic gauging (FDG) cross-flow filtration system.

The filtration test rig with integrated FDG system is illustrated in Figure 1. It consisted of a filtration cell with a 150 × 16 mm flat sheet membrane placed on a support of porous polypropylene. A gear pump (MCP-Z, Ismatec, Wertheim, Germany) fed the cell with feed from a feed tank, in which the feed suspension was stirred. Between the pump and the flow cell, an air dampener was placed to reduce pulses from the pump. The transmembrane pressure (TMP) was controlled by a downstream needle valve and measured with a pressure transducer (PXM419-010BA10V, Omega Engineering Ltd, Manchester, UK), which was connected to a PC for data collection. Retentate was circulated back to the feed tank and permeate was collected in a beaker on a balance (PB3002, Mettler Toledo, Columbus, OH, USA), also connected to a PC for data collection.

**Figure 2:**
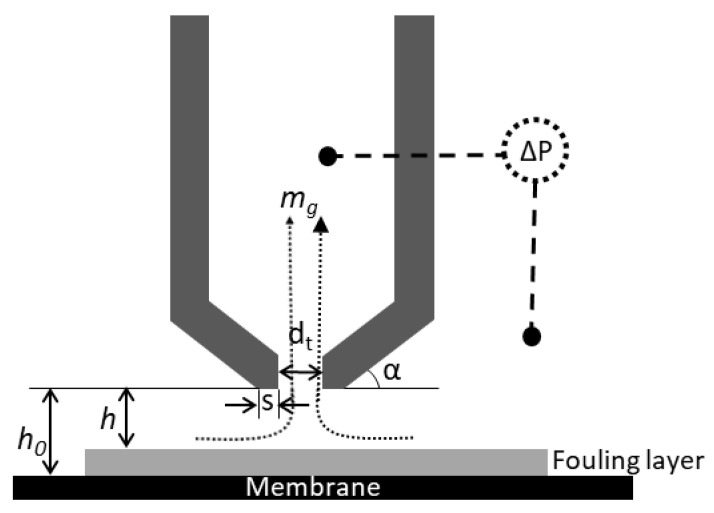
Schematic diagram of the FDG probe over a fouled membrane surface. The probe used in this study had the following dimensions: inner diameter of the nozzle, dt, was 0.5 mm, while the nozzle rim, s, was 0.25 mm, and α = 45°. mg is the gauge flow, and ΔP is the pressure drop over the probe, while *h*_0_ and *h* are the clearing heights over the membrane and the fouled membrane, respectively.

The FDG gauge probe (Figure 2) was mounted in the centre of the filtration cell. The inner diameter of the nozzle was 500 µm, and the FDG measurements affected an area of about 1 × 1 mm. The gauge flow was generated using gear pump (DBS.11EEET2NMM104, Tuthill, Alsip, IL, USA) and regulated using a mass flow controller (Mini CORI-FLOW, Bronkhorst, Ruurlo, NL). In this study, a constant gauge flow, mg, of 0.1 g/s was applied. The pressure drop over the probe was measured with a differential pressure transducer (PX419-2.5DWUV, Omega Engineering Ltd, Manchester, UK) and logged in a LabView software on the PC. A stepper motor (Nanotec ST4209S1006-B, Nanotech Electronic GmbH, Feldkirchen, DE) was used to move the position of the gauge probe stepwise with a minimum increment of 0.3 µm, and the probe position was monitored with a potentiometer (534, Vishay Spectrol, Malvern, PA, USA) and a linear variable differential transformer (LVDT, SM-series LVDT, Singer Instruments, Tirat Carmel, IL, USA). The LVDT enabled monitoring of the probe’s position with an accuracy of ± 0.5 µm; the potentiometer’s accuracy was lower, however it was used during long ranged movements, allowing the probe to be withdrawn from the vicinity of the membrane surface to avoid interference during fouling layer build-up. The calibration of the relationship between pressure drop over the probe and the distance to a flat surface is described in Zhou et al. [12], and calibration data from this study were used in the present study. The following equation, Equation (1), was fitted to measured gauge pressure drop (Δ*P*) and dimensionless distance from the surface of a metal plate (*h*_0_/*dt*) for *h*_0_*/dt* ≤ 0.25 using collected measure data up to Δ*P* = 99.3 mbar [12].

(1) $$\Delta P=c_{1}e^{\frac{c_{2}}{h/dt}}+c_{3}e^{\frac{c_{4}}{h/dt}}$$

Fitting the function to the measured data gave the following values for the constants: *c*_1_ = 1135.34 mbar, *c*_2_ = −1.57, *c*_3_ = 2.58 mbar, and *c*_4_ = 0.302 [12].

### 2.3. FDG Filtration Experiments

The 3 L feed suspensions were prepared for filtration experiments using deionised (DI) water, NaOH, and synthesised microparticles. The concentration of synthesised microparticles was 0.02 (v/v%), and NaOH concentration was 5 mM. Four types of suspensions were prepared in all: two suspensions following the procedure as mentioned above with either LC or HC particles (denoted LC or HC w/o Ca) and two suspensions (LC and HC, respectively) with 7.5 mM CaCl_2_ along with 5 mM NaOH (denoted LC or HC w/ Ca). For LC and HC w/ Ca, three individual filtration experiments were conducted for each of the suspensions, and for LC and HC w/o Ca, two filtration experiments were conducted for each of the suspensions. FDG was used to characterise the properties of the resulting fouling layer in each experiment.

The membrane used for the filtration experiments was a regenerated cellulose microfiltration (MF) membrane with a nominal pore size of 0.2 µm (RC58, GE Whatman, Little Chalfont, UK), which is a commonly used MF membrane with a high hydrophilicity. The membrane surface charge was analysed by streaming potential measurement (Anton Paar SURPASS streaming potential analyser, Anton Paar GmbH, Graz, AT) and was measured to be −66.6 ± 3.9 mV in 5 mM NaOH and −24.4 ± 2.8 mV in 5 mM NaOH and 7.5 mM CaCl_2_. In a previous study, the membranes were characterised with AFM showing a surface roughness with an arithmetic mean of 127 ± 9 nm and a root mean square roughness of 156 ± 36 nm [12]. An AFM height image of the pristine membrane can be found in Zhou et al. [12]. The effective membrane area in the filtration cell was 0.0024 m^2^. A fresh, pre-soaked membrane was used for each filtration experiment. During all filtrations, the feed pump was adjusted to a flow rate of 89.6 L/h, resulting in a cross-flow velocity in the filtration cell of 0.1 m/s, which corresponds to a wall shear stress of 0.045 N/m^2^ and a Reynold’s number of Re = 1630. TMP was adjusted to 400 mbar.

Before each fouling experiment, a DI water filtration was performed with the membrane mounted in the filtration cell. During the filtration, a scan (denoted “membrane scan”) was conducted to determine the position of the membrane. During the scan, the probe was moved towards the surface, and the resulting pressure drop over the FDG probe was recorded along with pure water flux. Then, the microparticles, NaOH and, for experiments w/ Ca, CaCl_2_ were added to the suspension, and the membrane was fouled for 1000 s, during which the mass of the permeate was also recorded to calculate permeate flux. During the fouling phase, the probe was withdrawn from the vicinity of the membrane surface, and the gauge flow was suspended. When 300 mL permeate had been collected, the permeate was returned to the feed tank to avoid concentrating the suspension. After 1000 s fouling, a scan (denoted “fouling scan”) was conducted again to determine the thickness and cohesive strength of the developed fouling layer. Finally, after resulting removal of the cake layer in the second scan, a third scan, “post scan”, was conducted to determine the remaining amount of fouling.

### 2.4. Analyses

After the filtration experiments, cake layers from the 150 × 16 mm membrane area were recovered by the procedure described in Zhou et al. [12] and dried in an oven at 105 °C. Subsequently, the dry cake was weighed to determine the specific mass of cake relative to membrane area. Suspensions were analysed by zeta-potential measurement (Zetasizer nano ZS, Malvern Instruments, Malvern, UK) to determine an average zeta potential out of three measurements. In addition, samples were analysed by size measurement (Model 13320, Beckman Coulter, Brea, CA, USA).

The permeate flux, *J*, was calculated from the rate of increase in mass of the permeate beaker, Δ*m/*Δ*t*, as described in Equation (2):

(2) $$J=\frac{\Delta m}{\Delta t}\frac{1}{A\cdot \rho_{w}}$$

where *A* is the membrane area and *ρ_w_* is the permeate density.

The scans of gauge pressure drop vs. probe position near the clean and fouled membrane gave pressure drops at different gauge positions. The measurements for the clean membranes were related to the calibration profile to determine offsets due to, e.g., differences in membrane mounting position and static pressure differences. These offset values were then applied to both the “fouling scan” and “post scan” data sets. Then, using Equation (1), clearing heights before (*h*_0_) and after fouling (*h*) could be determined from the measured pressure drops. Only *h*/*dt* < 0.20 data were used in the following analysis, due to a low response in pressure drop at higher *h/dt* values.

Following this, the thickness of the cake layer, *δ*_c_, could be estimated by comparing the clearance height over a clean membrane, *h*_0_, and over a fouled membrane, *h*, according to Equation (3):

(3) $$\delta_{c}=h_{0}-h$$

To assess the cohesive strength of the fouling layer, the maximum shear stress directly below the inner edge of the nozzle rim, *τ*_w,max_, was estimated. This was conducted assuming a creeping concentric flow between parallel plates, i.e., between the nozzle and surface, an approach that has been used in earlier work [12,20] and has been found to agree with CFD 2D simulations for estimation of *τ*_w,max_ [30]:

(4) $$\tau_{w,max}=\mu \left(\frac{6m_{g}}{\rho \pi h^{2}}\right)\frac{1}{d_{t}}$$

In the equation above, *µ* is the liquid’s dynamic viscosity, *m_g_* is the mass flow rate through the gauge, and *ρ* is the liquid density. Water properties at 22 °C were used to describe the liquid’s dynamic viscosity and liquid density.

## 3. Results

### 3.1. Characterisation of the Suspension

The characteristics of the suspensions, measured after filtration experiments, in terms of surface charge and particle (agglomerate) diameter (D10, median and D90 values), are presented in Table 1. The listed zeta potentials are averaged and reported together with standard deviations (triplicate runs for suspensions with calcium ions and duplicate runs for suspensions without calcium ions). All suspensions had a pH of 11.5 ± 0.1, and pH was not affected by the addition of CaCl_2_ salt.

The zeta potential of the particles in the suspensions with only Na^+^ cations, measured after filtration experiments, was similar to that of the particles after synthesis (−42 and −28 mV for HC and LC, respectively), i.e., the Na^+^ ions from NaOH did not reduce the particles’ zeta potential. For suspensions containing both Na^+^ and Ca^2+^ cations, the zeta potential was reduced to −5.6 ± 0.6 mV and −3.7 ± 0.5 mV. This is explained by the higher charge neutralisation by the (divalent) calcium ions compared to the (monovalent) sodium ions, because the divalent cations have a higher distribution closer to the Stern layer compared to the monovalent cations.

**Figure 3:**
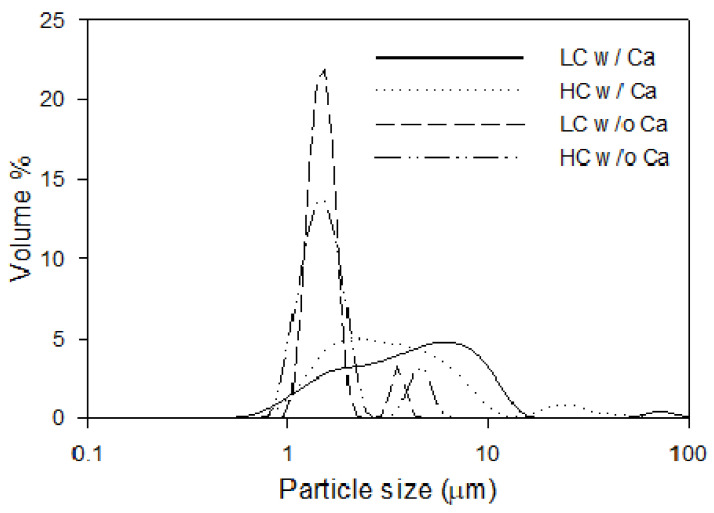
Volume-weighted size distribution of high surface charge (HC) and low surface charge (LC) PS–PAA particles with and without calcium ions present.

The measured particle size distributions are presented in Figure 3. Size measurements of the microparticles after filtration experiments with the presence of only Na^+^ cations were similar to those of the particles after synthesis with average diameters of 1.54 ± 0.34 µm and 1.54 ± 0.23 µm for HC and LC particles. However, there are signs of some coagulation; there were also peaks around 4.75 ± 0.61 µm and 3.71 ± 0.32 µm for HC and LC microparticles. For filtration suspensions with Na^+^ and Ca^2+^ ions, it can be observed from Figure 3 that the particles agglomerate because there was a shift in particle size. The median diameter was 4.43 µm for suspensions of HC particles in the presence of Ca^2+^, compared to 1.61 µm in the absence of Ca^2+^. For suspensions of LC particles, the agglomerate median diameter was 3.22 µm in the presence of Ca^2+^, and 1.55 µm in its absence. Another explanation for the coagulation observed for samples with Ca^2+^ present could be the elevated ionic strength of the samples. However, as shown in Appendix B, characterisation of samples with elevated ionic strength by the addition of monovalent Na^+^ ions did not show coagulation. Hence, the coagulation is described by the bridging effect of Ca^2+^ ions.

### 3.2. Formation of Cake Layers

**Figure 4:**
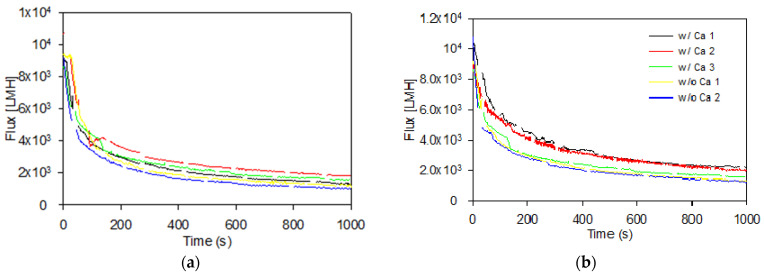
Decline in relative flux during 1000 s membrane fouling by filtration of low surface charge (**a**) and high surface charge (**b**) PS–PAA particles with and without calcium ions. Line breaks in the curves are caused by emptying the permeate beaker.

The permeate flux was obtained from recording the permeate flow during filtration as described in Section 2.2 and calculated using Equation (2). The flux was stable during the membrane scan prior to fouling, with an average pure (DI) water flux of 9509 ± 793 L/m^2^/h (LMH). As the microparticles were added along with NaOH and CaCl_2_, a rapid decline in flux was observed, and after filtration, a cake layer was observed and recovered from the membrane surface. During mechanical cake recovery after filtration, it was observed that the cakes formed in the presence of calcium appeared stronger than cakes formed in the absence of calcium ions. The latter were more difficult to recover due to their instability. The instability of the cakes formed in suspensions without calcium ions was attributed to charge repulsion between the negatively charged particles as well as charge repulsion between the particles and the negatively charged membrane (streaming potential −66.6 ± 3.9 mV in 5 mM NaOH). In the presence of calcium ions, the membrane streaming potential was reduced to −24.4 ± 2.8 mV, resulting in less repulsion between membrane and microparticles. The flux vs. time profiles during the 1000 s filtration phases are shown in Figure 4.

Comparing the flux profiles in Figure 4 shows that the overall flux decline behaviour was similar between LC and HC PS–PAA microparticles with and without Ca^2+^, with a somewhat more pronounced flux decline occurring for the suspensions without added Ca^2+^. The flux decline percentage during 1000 s filtration of LC and HC PS–PAA microparticles with and without Ca^2+^ is shown in Table 2, along with the specific mass of cake recovered after filtration and FDG scans. This generally shows a higher relative flux decline during the 1000 s filtration of particles in the absence of calcium (87.2 ± 2.3% and 88.6 ± 0.6% for HC and LC particles, respectively), compared to 1000 s filtration of suspensions in the presence of calcium ions (80.0 ± 1.8% and 81.2 ± 2.2% for HC and LC particles), where also a larger average particle size was observed (Figure 3). The specific cake masses recorded are somewhat higher for recovered cake layers formed in the presence of calcium ions, compared to those formed without calcium ion addition. This could indicate a difficulty of fully recovering potentially weaker cakes formed in the absence of calcium. The large standard deviations of the specific cake masses make it difficult to conclude that there are substantial differences in the mass of cake layer between the different filtration conditions.

### 3.3. FDG Analysis of Cake Fouling Layers

**Figure 5:**
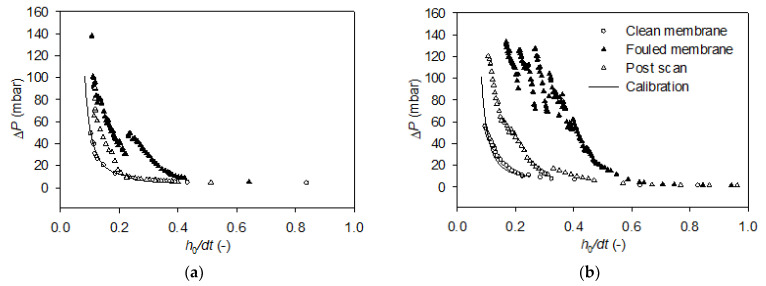
FDG pressure drop vs relative distance, *h*_0_*/dt*, profiles for fouling experiments with low surface charge (**a**) and high surface charge (**b**) PS–PAA microparticles with calcium ions. Clean membrane measurements have been used to determine offsets which have been applied on the “fouled membrane” and “post-scan” data series.

Membranes were scanned with the FDG probe before and after filtration to determine the pressure drop at different probe distances from the surfaces. In Figure 5, representative curves of clean membranes and fouling layers formed by HC and LC particles in the presence of Ca^2+^ ions are shown, along with post-scans after fouling layer removal by the probe. The pressure drop data from the additional FDG experiments can be found in Appendix A.

For the clean membrane, the scans show a low, constant pressure drop at *h*_0_*/dt* > 0.3 (approx. 10 mbar). As the probe was moved closer to the surface, *h*_0_/*dt* decreased, and the pressure drop increased exponentially. During scans of the fouling layer, the elevation in pressure drop started earlier, i.e., at a higher *h*_0_*/dt* value. This means that, at the same position, the probe was closer to a surface than at the previous membrane scan, thus a deposit had formed on the membrane. As the probe was moved closer to the surface, Δ*P* did increase, but not with the same slope as for the clean membrane. This indicates a continuous removal of cake by the fluid shear stress generated from the probe as it moved further towards the surface.

As *h*_0_/*dt* was reduced further, a distinct drop in Δ*P* can be observed in Figure 5a. This corresponds to the removal of a larger piece of the cake layer by the high shear force, increasing the distance between the probe and the remaining fouling layer.

The post-scan, which was performed after the destructive fouling scan, is also plotted in Figure 5a. This scan shows a Δ*P-h*_0_*/dt* profile resembling that of the clean membrane at *h*_0_*/dt* > 0.20. At *h*_0_*/dt* < 0.20, the pressure drop was higher at the same positions. By use of this scan, the thickness of residual fouling layer can be determined at varying shear stresses applied by the gauge flow. At a maximum shear stress of *τ*_w,max_ = 100 N/m^2^, the thickness of remaining fouling layer was determined to be 23.3 µm. Repeating this analysis for the remaining LC scans showed an average remaining thickness of 28.8 ± 28.4 µm for the three scans with Ca^2+^ present, whereas it was only 3.2 ± 1.9 µm for the cakes formed without presence of Ca^2+^. This is explained by the repulsion between negatively charged particles as well as between particles and the membrane in the absence of Ca^2+^ and charge shielding and bridging effect in the presence of the Ca^2+^. The reason for the high standard deviations is that some of the cakes were completely removed.

The scan of the fouling layer formed by HC particles with Ca^2+^ presented in Figure 5b shows an elevation in pressure drop, reaching Δ*P* = 20 mbar already at *h*_0_*/dt* = 0.49. Hence, the probe detects a surface located farther from the membrane for the fouling layer formed with HC particles, compared to LC particles. This is a general tendency observed for the three FDG scans of HC and LC particles with calcium ions, see Appendix A. In Figure 5b it can be observed that as the probe moved closer towards the membrane, the pressure drop increased gradually and reached 102 mbar at *h*_0_/*dt* = 0.34. After a further reduction in *h*_0_*/dt*, Δ*P* dropped to 69 mbar, indicating the removal of a larger fraction of cake layer. Further reductions in *h*_0_*/dt* led to gradual elevations in Δ*P*, followed by drops as Δ*P* exceeded critical values in the 102–122 mbar range. The destructive FDG scan of the fouling layer in Figure 5b was stopped at Δ*P* = 131 mbar, and a post-scan was employed to determine the degree of irremovable fouling. This scan showed lower pressure drops at the same positions as the fouling scan, confirming some cake removal. However, a comparison with the clean membrane profile suggests that there was still a deposit of HC particles on the membrane, not removed by the probe. Again, this scan was used to determine the thickness of residual fouling layer. At a maximum shear stress of *τ*_w,max_ = 100 N/m^2^, the thickness of remaining fouling layer was estimated to be 56.1 µm for the scan presented in Figure 5b. The average thickness was 65.5 ± 28.5 µm for cakes formed in the presence of Ca^2+^ and 7.6 ± 5.3 µm for cakes formed in the absence of Ca^2+^. Thereby, the fouling layer formed by HC particles displayed a higher cohesivity and was less removable than cakes formed by LC particles, which is explained by the higher charge density promoting bridging between particles and between particles and membrane. It was also found that the thickness of cakes formed by HC particles in the absence of Ca^2+^ were slightly thicker than cakes formed by LC particles in the absence of Ca^2+^. This was not expected, because a higher charge density would be expected to increase repulsion and reduce cake formation.

**Figure 6:**
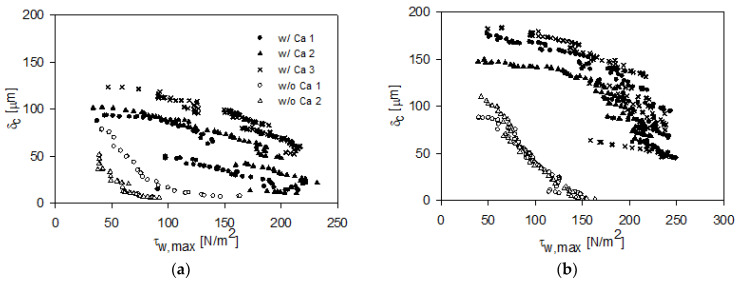
Plot of estimated cake height vs. applied maximum shear stress for fouling layers formed by low surface charge (**a**) and high surface charge (**b**) PS–PAA microparticles in the presence and absence of calcium ions. Shear stresses above 214 N/m^2^ were based on *h*-values estimated using an extrapolation of Equation (1).

A deeper analysis of the cohesive strength of the cake fouling layers was carried out by calculating the maximum shear stress applied by the probe, τ_w,max_ (Equation (4)), at varying residual cake thicknesses, estimated by Equation (3). The results for the three FDG scans of HC and LC particles with Ca^2+^ and the two FDG scans of cakes from HC and LC particles without Ca^2+^ are plotted in Figure 6. It should be noted that values of *τ*_w,max_ > 214 N/m^2^ were calculated from *h*-values estimated by extrapolation above the calibration span of Equation (1). All shear stresses applied by the gauge flow exceeded the shear stress induced by the cross-flow (0.045 N/m^2^), thus, the contribution by cross-flow on the removal of fouling during the FDG scans were negligible compared to the shear stresses induced by the gauge flow.

Figure 6a shows the estimated thicknesses of cakes formed by LC particles with and without calcium. At an applied shear stress of *τ*_w,max_ = 50 N/m^2^, the estimated residual thicknesses of the cakes formed without calcium ions were within the 24–69 µm range, whereas cakes formed in the presence of Ca^2+^ were within the 92–123 µm range, hence they required more shear to be removed. As cake was removed and cake thickness reduced, a higher shear stress was required to remove the remaining cake, i.e., the gradual decline in the *δ*_c_ − *τ*_w,max_ profile illustrated how more liquid shear stress was required to remove the lower layers, which was a result of higher cohesive strength at the bottom of cakes than at the top of the cakes as seen in previous studies [12,22]. This observation is explained by the compressibility of the cake resulting in denser structures near the membrane in the bottom of the cake.

For highly charged particles, the FDG scans (Figure 6b) again showed lower cohesive strength of cakes formed in the absence of calcium. The initial amount of cake detected was 88–100 µm at *τ*_w,max_ = 50 N/m^2^ and was almost completely removed at *τ*_w,max_ = 160 N/m^2^. The estimated thickness of HC cakes formed in the presence of Ca^2+^ was larger (146–182 µm at *τ*_w,max_ = 50 N/m^2^) than for cakes formed without calcium or with LC particles. This again demonstrates the contribution of ionic bridging between charged particles to cohesive cake strength. As the probe scanned through the layer, reducing *δ*_c_, critical points for the removal of larger fractions of cakes are reached, which for the cakes formed by HC particles with Ca^2+^ were in the *τ*_w,max_ = 194–244 N/m^2^ range. However, for the HC particles deposited with calcium ions, not all cake layer was removed by the destructive FDG scans. Analysis of the post-scan, reported above, also showed that the removability of cake layers formed in the presence of calcium ions was lower if they were formed by highly charged surface particles, compared to lower charged particles. These results are supported by the zeta potential measurements of the membrane surface presented in Section 2.3: FDG filtration experiments and of HC and LC particles in Table 1, showing a reduction in zeta potential with the addition of Ca^2+^. This will affect the deposition, because larger agglomerates form, but also the cohesiveness and removal because there is less electrostatic particle–particle repulsion and particle–membrane repulsion.

The results of this study demonstrate how FDG can be a valuable tool to quantify and understand the effects of physico-chemical conditions on cake cohesive strength and removability. The method has already proven its merits in different applications, such as characterising fouling layers formed in the filtration of yeast, microcrystalline cellulose, biofilms, etc. Therefore, it is relevant to prove the merits for other applications, e.g., brewing and dairy industries and in water and wastewater treatment.

## 4. Conclusions

Cross-flow filtration experiments were performed on synthetic core-shell microparticles with varying surface charge and counter ions in solution. FDG was applied to quantify and study the cohesive strength of the fouling layers formed. It was demonstrated that FDG can be applied to assess the difference in cohesive strength between fouling layers formed by negatively charged particles in the presence and absence of calcium ions and it was demonstrated that the interactions between negatively charged particles and calcium ions resulted in cohesive fouling layers, whereas fouling layers formed in the absence of calcium ions were easily removed by the applied liquid shear stress. In line with this, it was found that fouling layers formed by particles of high negative charge (−42 mV, in the presence of calcium) had higher cohesive strength than fouling layers formed under the same conditions by lower negatively charged particles (−28 mV).

Finally, it was shown that after an implementation of a post-scan after the destructive FDG scans of the cake, the remaining amount of cake on the membrane could be estimated to describe the irremovability of cake layers formed under different conditions.

## Figures and Tables

**Table 1:** Zeta potential and diameters (D10, median and D90 values) of particles in suspensions after filtration. The suspensions were formed by suspending higher and lower charged PS–PAA microparticles in DI water together with NaOH (w/o Ca) or NaOH as well as CaCl_2_ (w/ Ca), respectively.

	Zeta Potential (mV)		Particle Diameter (µm)					
	5 mM NaOH	5 mM NaOH + 7.5 mM CaCl_2_	5 mM NaOH			5 mM NaOH + 7.5 mM CaCl_2_		
			D10	Median	D90	D10	Median	D90
Low Charge	−30.6 ± 1.0	−3.7 ± 0.5	1.27	1.55	1.97	1.45	3.22	8.76
High Charge	−39.4 ± 0.9	−5.6 ± 0.6	1.27	1.61	4.39	1.45	4.43	10.17

**Table 2:** Specific mass of recovered fouling layer after filtration and relative flux decline during 1000 s filtration of LC and HC suspensions with and without calcium ions.

Variable	LC w/ Ca	LC w/o Ca	HC w/ Ca	HC w/o Ca
Specific cake mass (g/m^2^)	157 ± 15	141 ± 49	153 ± 29	137 ± 23
Relative flux decline	81.2 ± 2.2%	88.6 ± 0.6%	80.0 ± 1.8%	87.2 ± 2.3%

**Figure A1:**
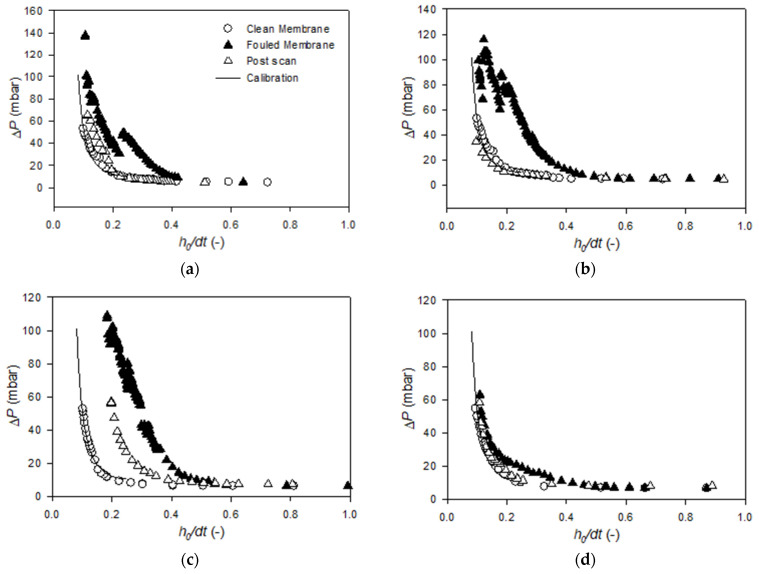

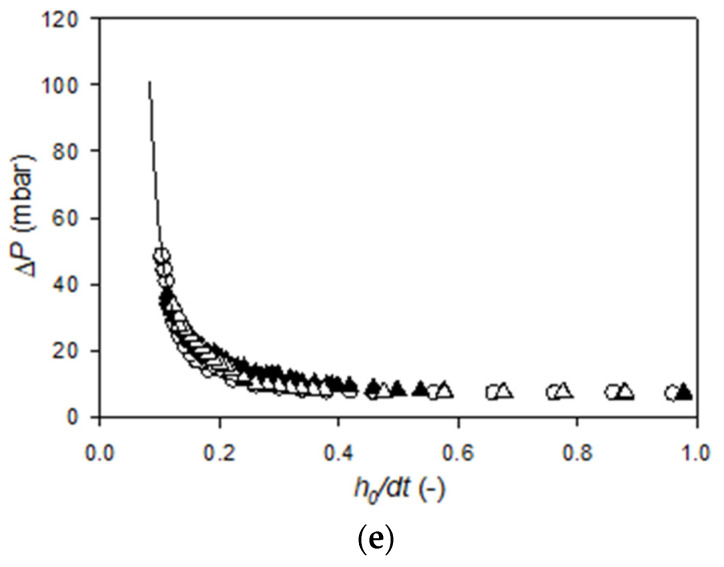
Profiles of measured FDG pressure drop vs relative distance, *h*_0_/*dt*, for fouling experiments with low surface charge PS–PAA microparticles with (**a**–**c**) and without (**d**,**e**) calcium ions.

**Figure A2:**
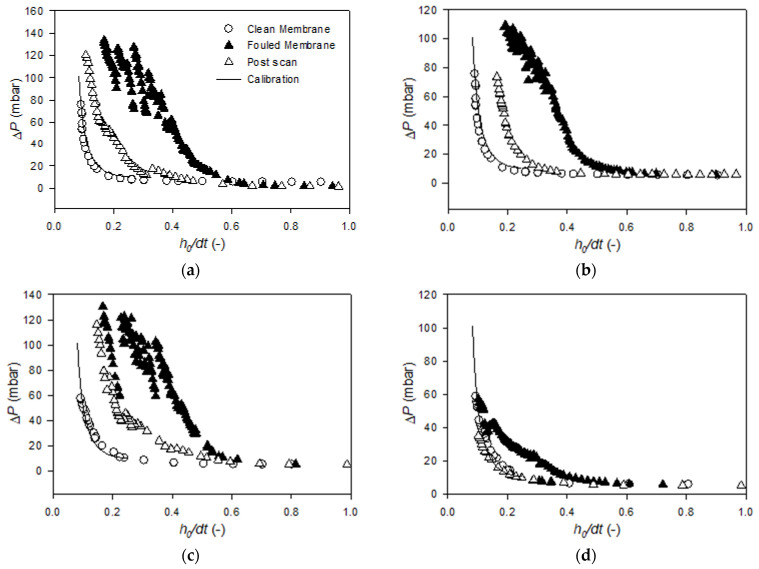

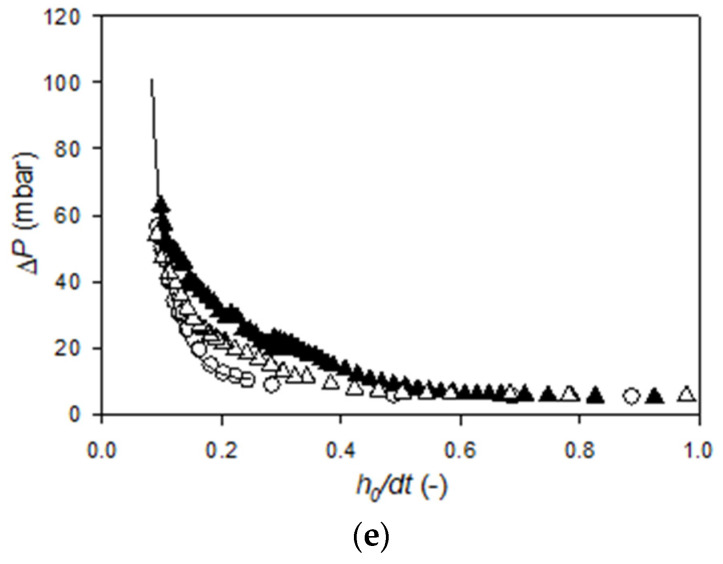
Measured FDG pressure drop vs relative distance profiles for fouling experiments with high surface charge PS–PAA microparticles with (**a**–**c**) and without (**d**,**e**) calcium ions.

## Data Availability

The data presented in this study are available on request from the corresponding author.

## Appendix A

All FDG profiles from experiments with low surface charge microparticles with and without Ca^2+^ are shown in Figure A1. The profiles include a scan of FDG pressure drop vs. relative distance for a clean membrane before fouling, a fouled membrane, and a post-scan after the membrane scan. The calibration curve is also plotted.

Figure A2 shows profiles of FDG pressure drop vs. relative distance for FDG scans from filtration experiments with high surface charge microparticles with and without Ca^2+^. As in Figure A1, scans of clean membrane, fouled membrane and as well as post-fouling removal scans are shown.

## Appendix B

To determine whether the coagulation of negatively charged LC and HC particles was induced by ionic strength rather than the addition of divalent, bridging, cations (Ca^2+^ from 7.5 mM CaCl_2_ in 5 mM NaOH), suspensions with similar ionic strength, but composed of 22.5 mM NaCl (instead of CaCl_2_) in 5 mM NaOH were characterised by zeta potential and particle size measurements with the methods described in Section 2.4. The measured zeta potentials and size distributions are shown in Table A1.

**Table A1:** Zeta potential and diameters (D10, median and D90 values) of low and high charge particles suspended in 5 mM NaOH and in 22.5 mM NaCl with 5 mM NaOH.

	Zeta Potential (mV)		Particle Diameter (µm)					
	5 mM NaOH	5 mM NaOH + 22.5 mM NaCl	5 mM NaOH			5 mM NaOH + 22.5 mM NaCl		
			D10	Median	D90	D10	Median	D90
Low Charge	−30.6 ± 1.0	−27.7 ± 0.8	1.27	1.55	1.97	1.15	1.39	1.63
High Charge	−39.4 ± 0.9	−38.7 ± 1.9	1.27	1.61	4.39	1.11	1.58	2.05

The results show that there was no significant reduction in zeta potential by the elevated ionic strength, neither does it show an increase in particle sizes.
